# Maternal MUAC and fetal outcome in an Indian tertiary care hospital: A prospective observational study

**DOI:** 10.1111/mcn.12902

**Published:** 2019-12-12

**Authors:** Donugama Vasundhara, Rajkumar Hemalatha, Saurabh Sharma, Baru Anantha Ramalaxmi, Varanasi Bhaskar, JagJeevan Babu, Radha Krishna Kankipati Vijaya, RajaSriswan Mamidi

**Affiliations:** ^1^ National Institute of Nutrition (ICMR) Jamai Osmania Hyderabad India; ^2^ National Institute of Medical statistics (ICMR) New Delhi India

**Keywords:** Birth Weight, Fetal outcome, Gestational weight gain, Low birthweight, MUAC, Pregnancy

## Abstract

Studies to date demonstrated the relatedness of mid‐upper arm circumference (MUAC) measurement of pregnant women to their anthropometry/weight. Hence, the objective was to determine whether maternal MUAC at different gestational age predicted birthweight, and if so, to identify which cut‐offs provided the best prediction of low birthweight (LBW) in pregnant women cohort. A total of 928 pregnant women, free of any obstetrical and medical complications known to affect fetal growth, were followed from 20 to 24 weeks' gestation till delivery. Weight, height, and MUAC were determined for the pregnant women, and gestational age along with newborns anthropometry was collected. The mean birthweight was 2.6 ± 0.460 kg. Maternal age, height, weight, MUAC (three time points), gestational age at delivery, and post‐natal weight showed positive correlation with birthweight, crown heel length, and head circumference of the neonates. The cut‐off limit with the best sensitivity–specificity (54.0 and 59.8, respectively) for MUAC was 23 cm, whereas maternal weight of 55 kg had sensitivity and specificity of 62.5 and 59.9 for predicting LBW. Maternal weight of 55 kg and MUAC value of 23 cm had almost similar sensitivity and specificity for predicting LBW. MUAC (≤23 cm) can be considered as a potential indicator of LBW where weighing of pregnant women is not feasible or when presentation for antenatal care is late, especially where pre‐pregnancy weights are not available.

**Key messages**

- Though MUAC is identified as a marker to assess nutritional status of pregnant women by WHO, data on optimal cut‐off points are lacking especially in Indian context.
- This is the first study to demonstrate relationship between MUAC and maternal weight gain on three serial measurements.
- We found a similar sensitivity–specificity of MUAC for predicting LBW to that of maternal weight, suggesting the validation of MUAC.
- Contributing to validate cut‐off point of MUAC measurements to assess poor pregnancy outcomes, which can help in identifying women at risk.

## 1 INTRODUCTION

Low birthweight (LBW) is recognized as an important determinant of neonatal mortality and morbidity, and it is one of the World Health Assembly targets by 2025. The annual average rate of reduction in LBW prevalence globally was only 1.2% between the year 2000 and 2015 (UNICEF‐WHO LBW estimates, 2019). To affect the future survival of the newborn and to improve quality of adult life, neonatal birthweight (BW) should be improved, which in turn is directly dependent on maternal anthropometry (Sen, Roy, & Mondal, 2009). Considerable attention has been focused on the maternal anthropometric measurements as indicators of LBW for identifying women at risk of LBW. Impaired nutritional status of women before conception, short stature, and poor nutrition during pregnancy are important contributing factors of LBW (Sen et al., 2009; Muthayya, 2009; WHO‐Provisional agenda item 6.3, 2011).

Maternal anthropometry such as maternal weight (Wt), height (Ht), mid‐upper arm circumference (MUAC), and maternal body mass index (BMI) in the first trimester are suggested as good predictors of LBW. Nevertheless, pre‐pregnancy body mass index and gestational weight gain are the preferred anthropometric indicators to identify women at risk of producing LBW babies. However, in India, pregnant women begin to visit antenatal clinics after 10 to 12 weeks of pregnancy, and therefore, pre‐pregnancy weight may not be available to calculate BMI. Considering various influencing factors, studies have demonstrated that MUAC is closely related to maternal weight, and therefore, it has been suggested as an effective tool for maternal nutrition status screening (Elshibly & Schmalisch, 2008; Tang et al., 2016; Lechtig, 1988). However, knowledge about its changes during the course of pregnancy and the cut‐off that could predict LBW is limited (Frison, Kerac, Checchi, & Prudhon, 2016). There is lacuna of established cut‐off points of maternal MUAC by taking demographic differences into account. MUAC of <22 cm has been suggested as an indicator of wasting, but MUAC cut‐off points to predict LBW are suggested to be different for different regions (Villamor et al., 2002). Yet most studies have measured MUAC just before or after delivery; data on changes in MUAC values during pregnancy are limited (Tang et al., 2016; Ojha & Malla, 2007; Dhar & Bhadra, 2008; Sebayang et al., 2012; Assefa et al., 2012; Shrivastava, Agrawal, & Giri, 2016; Mohanty et al., 2005; Ricalde, Velásquez‐Meléndez, Tanaka, & de Siqueira, 1998; López, Calvo, Poy, del Valle Balmaceda, & Cámera, 2011). Ricalde et al. collected three serial measurements on 92 pregnant women in Brazil, and Lopez et al. determined pattern of changes in MUAC, triceps, biceps, and subscapular skinfold thicknesses during the course of pregnancy. In 2016, WHO has recommended that MUAC may be useful to identify undernourished pregnant women and suggested that the optimal cut‐off points must be determined for individual countries based on context‐specific cost‐benefit analyses (World Health Organization‐Recommendations on antenatal care, 2016). Keeping in view the above, the current prospective cohort study determined maternal MUAC at different gestational age and also assessed the sensitivity of predicting women at risk of LBW.

## 2 METHODS

### 2.1 Study setting

In a prospective cohort study, pregnant women who received antenatal care at a tertiary care government maternity hospital located at Hyderabad, Telangana, and were delivered at the same hospital from the period May 2012 to May 2015 were screened.

### 2.2 Sample size calculation

Assuming a sensitivity of 60% with a similar specificity of 60%, taking a precision of 10% and 95% confidence interval (CI) with 80% power the sample size required was 556. However, expecting attrition of 35% due to long follow‐up, the sample size was calculated to 751. But 928 were found to be eligible and were recruited for screening (Nahar, Mascie‐Taylor, & Begum, 2007; Roy & Sen, 2018).

### 2.3 Participants

**Figure 1:**
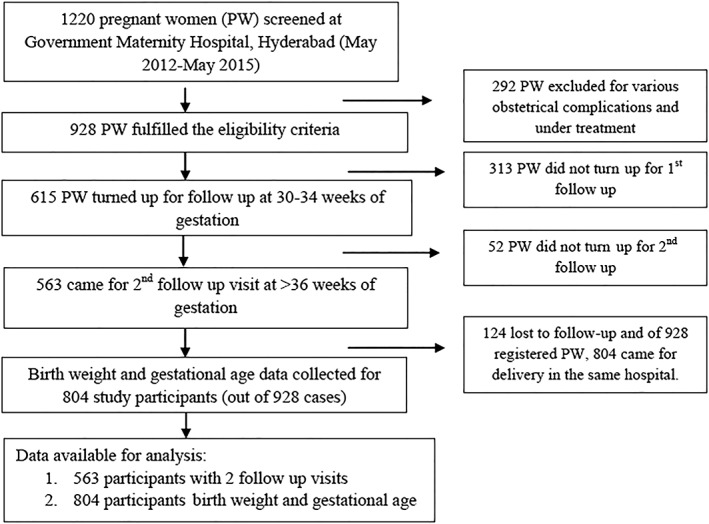
Recruitment flow diagram

Pregnant women in their first and second trimester, willing to participate in the study, were included for the recruitment. Women with gestational age nearing 30 weeks, gestational diabetes, severe anaemia, pre‐eclampsia, chronic hypertension, fetal anomaly, rheumatoid arthritis, thyroid and parathyroid disorders, and hepatic or renal or cardiovascular diseases were excluded from the study. Nine hundred and twenty eight (928) pregnant women who fulfilled inclusion criteria were registered after obtaining written informed consent and were followed through during pregnancy till child birth. Of the 928, 615 and 563 turned up for follow‐up during 30–34 weeks' and >36 weeks' gestation, respectively; but delivery data such as BW and gestational age were collected from 804 of 928 recruited women. Only singleton deliveries were included in the final analysis of the data (Figure 1). In India, various programmes like “Janani Suraksha Yojana” under the National Rural Health Mission are functional under which women are paid a substantial fund for each ANC (antenatal check) visit to encourage ANCs and institutional deliveries. This enabled us to get good follow‐up of the study cohort. In the state where the study was conducted, 74.9% of pregnant women had four antenatal care visits to the health centers (Fact sheets‐2015‐16, NHFS‐4, India). All procedures performed in the study were in accordance with the ethical standards of the institutional and/or national research committee and with the 1964 Helsinki declaration and its later amendments.

### 2.4 Procedures

Anthropometric indicators include maternal weight in kg, height in cm, and MUAC in cm. BMI was calculated by taking pregnant women's weight in kg divided by her height in metres squared. BMI of less than 18.5 was classified as chronic energy deficiency or undernourished. Wt, MUAC, and skinfolds at four sites were collected at three time points during pregnancy (20–26 weeks', 30–34 weeks', and >36 weeks' gestation). MUAC was measured in the right arm at the level, midway between acromion and olecranon processes in centimetre, to the nearest decimal place. Triceps, biceps, and subscapular skinfold thickness were measured by trained nutritionists using a Lange skinfold caliper (nearest to 1 mm) according to standardized methods. The average of three measurements was recorded at each site. Maternal body composition was evaluated for all the three times during gestation. Post‐natal weight was collected after 24 hr but within 5 days after delivery. Two dedicated project staff, nutritionists were doing the anthropometry after due training and inter/intra rater reliability was done every 3 months to keep the CV below 10%. All the demographic and pregnancy details along with 24‐hr diet recall were collected from the participants.

The babies were examined within 24 hr of delivery, and BWs, crown heel length, and head circumference were recorded using the Seca weighing scales (to the nearest 1 g), infanto‐metre, and measuring tape (to the nearest 1 cm), respectively. As per the WHO (1995) definition, newborns weighing less than 2.5 kg were considered as LBW neonates. Gestational age was determined by dating the last menstrual period and at the time of recruitment and was corrected by first trimester ultrasonographic findings if the difference exceeded 5 days. SGA neonates were determined by comparing the BW with fetal growth standard by gestation week. Neonates with BW less than 10th percentile of the standard population for gestation week were considered as small for gestational age (Papageorghiou et al., 2014). SGA is a surrogate marker for identification of newborns with fetal growth restriction.

### 2.5 Statistical analysis

Data were analysed using SPSS 17.0 for windows IBM, Chicago. Mean and *SD* were calculated; mothers' weight, height, MUAC, and BMI were ascertained for a BW of 2.5 kg. Odds ratios were computed to assess the risk of LBW between various cut‐off points of MUAC and 95% CIs were calculated. Adjusted odds ratios from multivariable regression models were also performed. In addition, sensitivity, specificity, positive predictive value, negative predictive value, and area under the receiver operating characteristic curve (AUC) were calculated for predicting BW outcome for various MUAC cut‐offs. Pearson's correlation coefficients between longitudinal measurements of mothers' anthropometry and newborns' anthropometry were done. Significance considered at *P* value .05.

### 2.6 Ethical considerations

Institutional ethical committee approval was taken bearing the number 04/I/2014.

## 3 RESULTS

A total of 928 pregnant women were followed from 20 to 24 weeks' gestation till delivery. Mean age of the pregnant women was 21.9 ± 2.642, and 1.4% were less than 18 years age. A total of 17.8% of them were illiterate, 14.6% were moderate workers, and the rest being sedentary. However, all the study participants belonged to low socio‐economic status based on Kuppuswamy's guidelines (Bairwa, Rajput & Sachdeva, 2013). Mean energy (1,980 ± 829 kcal) and fat (33.8 ± 7.2 g) intakes were within recommended levels, whereas protein intake (54.66 ± 29.8 g) was lower than the recommended values of RDA for pregnant women. All the study participants were non‐smokers, non‐alcoholic, and free of any obstetrical and medical complications known to affect fetal growth. The mean ± *SD* gestational age at the time of recruitment was 23.6 ± 6.55. Of the 928 pregnant women followed, birth outcome data were collected from 804 pregnant women. Of the 804, 50.7% babies were males and the rest were females, and 73% delivered normally whereas 27% had caesarian section. The newborns were free of any congenital anomalies. The mean BW was 2.6 ± 0.460 kg. Frequency of LBW and preterm births (PTB) were 22.1% and 7.1 %, respectively, and 33% were SGA. One third (33%) of the babies were less than 10th percentile of the standard population (Papageorghiou et al., 2014) and were classified as SGA.

Maternal weight of the cohort ranged from 32 to 91 kg, merely 19.2 % were weighing more than 55 kg, and 44.6% were between 45 to 55 kg at the time of recruitment. A proportion of 36.2 % women in the study was weighing less than 45 kg. MUAC ranged from 16 to 35.5 cm; and 27.3 % were wasted when MUAC of less than 22 cm was considered. Height showed that 64.7 % had greater than 150 cm, and a proportion of 52.7 % had height greater than 152 cm. As for haemoglobin (Hb) status, only 23.2 % had Hb concentration more than 11 g/dl. Although 55.3 % had mild anaemia (9 to 11 g/dl), 18.4 % had moderate anaemia (7 to 9 g/dl), and severe anaemia was prevalent in 3.2% of pregnant women at the time of recruitment.

Mean values of weight and MUAC collected serially during three time points (20–24 weeks', 30–34 weeks', and >36 weeks' gestation) are given in Table 1. The mean height was 152.1 ± 5.606 cm, and the mean ± *SD* Wt was 48.6 ± 8.128 kg at the time of recruitment, which increased to 56.2 ± 8.788 kg by 38.5 ± 2.09 weeks of gestation. Over the same period, mean ± *SD* maternal MUAC increased from 23.7 ± 2.863 cm to 24.5 ± 2.866 cm. Predictably, mean age, height, weight, MUAC, and gestational age at delivery (GAD) were significantly low in LBW (Table 1). Post‐natal maternal weight was also low in LBW (Table 1). Likewise, Weight and MUAC during three time points were significantly low in SGA neonates (Table 2). Mean values of MUAC of pregnant women at 23 weeks gestation was 23.7 cm (±2.86), at 32 weeks 24.5 cm (±2.94), and at 38 weeks of gestation the MUAC was 24.5 cm (±2.86). MUAC was similar at different time points during pregnancy.

**Table 1:** Maternal and newborn anthropometry

	Total	Normal birthweight	Low birthweight	*P* value
Maternal parameters				
Age (804)	21.9 ± 2.642	22.0 ± 2.614 (626)	21.5 ± 2.484 (178)	.018
Height (cm; 804)	152.1 ± 5.606	152.6 ± 5.633 (626)	151.0 ± 5.255 (178)	.001
Maternal weight in kg at 23.6 ± 6.55 weeks' of gestation (804)	48.6 ± 8.128	49.7 ± 8.290 (626)	46.5 ± 8.260 (178)	.001
Maternal weight in kg at 32.6 ± 3.78 weeks' of gestation (615)	54.1 ± 8.554	55.2 ± 8.408 (482)	51.6 ± 9.042 (133)	.001
Maternal weight in kg at 38.5 ± 2.09 weeks of gestation (563)	56.2 ± 8.788	57.3 ± 8.715 (461)	52.5 ± 8.576 (102)	.001
MUAC (cm) at 23.6 ± 6.55 weeks of gestation (748)	23.7 ± 2.863	24.0 ± 2.916 (584)	23.2 ± 2.932 (164)	.001
MUAC (cm) at 32.6 ± 3.78 weeks of gestation (590)	24.5 ± 2.941	24.8 ± 2.926 (466)	23.7 ± 3.115 (124)	.001
MUAC (cm) at 38.5 ± 2.09 weeks of gestation (504)	24.5 ± 2.866	24.8 ± 2.825 (415)	23.3 ± 2.861 (89)	.001
HB (gm/dl) (629)	9.9 ± 1.447	9.9 ± 1.379 (492)	9.7 ± 1.571 (137)	.321
Gestational age at delivery (weeks; 748)	38.5 ± 2.090	38.9 ± 1.113 (624)	37.5 ± 2.475 (176)	.001
Post‐natal weight (kg; 611)	50.0 ± 8.796	50.6 ± 8.387 (482)	46.9 ± 8.444 (125)	.001
Newborn parameters				
Birthweight (kg)	2.6 ± .460	2.8 ± 0.315 (626)	2.04 ± 0.310 (178)	.001
Length (cm)	47.9 ± 2.221	48.3 ± 1.994 (463)	46.0 ± 2.099 (114)	.001
HC (cm)	32.7 ± 1.286	32.9 ± 1.163 (460)	31.7 ± 1.325 (109)	.001

*Note*. Values are mean ± *SD*. Values in parenthesis indicate number of pregnant women.

Abbreviations: HB, haemoglobin; HC, head circumference; MUAC, mid‐upper arm circumference.

**Table 2:** Mean and *SD* of maternal weight and MUAC during different stages of gestation in women with SGA babies

	Normal (≥10th percentile)	SGA (<10th percentile)	*P* value
Wt1, (804)	50.5±8.33	46.6±8.11	.001
Wt2, (615)	55.9±8.42	51.4±7.93	.001
Wt3, (563)	58.5±8.57	53.5±8.20	.001
MUAC1, (804)	24.3±2.94	23.2±2.74	.001
MUAC2, (615)	24.9±2.96	23.8±2.69	.001
MUAC3 (563)	25.1±2.96	23.7±2.49	.001

*Note*. Values are mean ± *SD*. Values in parentheses indicate number of pregnant women. Wt1 and MUAC1—data collected when mean and *SD* of gestation was 23.6 ± 6.55 weeks; Wt2 and MUAC2—data collected when mean and *SD* of gestation was 32.6 ± 3.78 weeks; and Wt3 and MUAC3—data collected when mean and SD of gestation was 38.5 ± 2.09 weeks.

Abbreviations: MUAC, mid‐upper arm circumference, SGA, small for gestation age; Wt, weight.

### 3.1 Mothers' weight, height, and MUAC values corresponding to BW

Table 3 shows the serial cut‐off values and validity indices of weight, height, and MUAC as an indicator of LBW. The best cut‐off limit with the highest sensitivity–specificity product for weight was 55 kg and height was 152 cm. Similarly, MUAC value of 23 cm had sensitivity and specificity of 54.0 and 59.8, respectively, whereas at 24 cm, the sensitivity rose to 71.3, but specificity decreased with an AUC value of 0.57. The mothers' weight, height, and MUAC values corresponding to BW of 2,500 g was calculated using the regression equation. The regression equation, 35.562 + 5.027 BW considering the mothers' weight corresponding to a BW of 2,500 g, was calculated to be 48.1 kg. Height was calculated to be 152.1 cm using the regression equation, 145.229 + 2.732 BW. Similarly, the maternal MUAC corresponding to a BW of 2,500 g was calculated to be 23.66 cm using the regression equation, 20.236 + 1.372 BW. Regression analysis of the LBW and the SGA neonates on Maternal MUAC1 with different cut‐offs showed 3.4 folds and 2.7 folds higher odds of having LBW or SGA neonates if the MUAC was less than 24 cm (Table 4).

**Table 3:** Serial cut‐off values and validity indices of maternal Wt, Ht, and MUAC as an indicator of LBW

Wt	Sensitivity	Specificity	PPV	NPV	Accuracy	AUC	*P* value
50	21.6	91.7	36.5	84.1	79.0	0.57	.005
55	62.5	59.9	25.7	87.8	60.4	0.64	.001
56	67.0	56.2	25.3	88.5	58.1	0.64	.001
57	71.6	51.1	24.5	89.0	54.8	0.63	.001
58	71.6	47.6	23.2	88.3	52.0	0.61	.005
Ht							
148	25.0	81.8	23.4	83.0	71.4	0.53	.320
150	39.8	70.1	22.9	83.9	64.6	0.55	.146
152	56.8	56.2	22.4	85.4	56.3	0.57	.056
154	69.3	41.0	20.7	85.7	46.2	0.55	.129
156	80.7	27.6	19.9	86.5	37.3	0.54	.224
MUAC							
20	12.6	96.2	42.3	83.3	81.0	0.54	.197
21	24.1	88.8	32.3	84.1	77.1	0.56	.059
22	36.8	74.8	24.4	84.2	67.9	0.56	.091
23	54.0	59.8	22.9	85.5	58.8	0.57	.044
24	71.3	42.0	21.4	86.8	47.3	0.57	.053
25	81.6	31.8	20.9	88.7	40.8	0.57	.050

Abbreviations: AUC, area under the curve; Ht, height; LBW, low birthweight; MUAC, mid‐upper arm circumference (Wt, Ht, and MUAC data of 23.6 ± 6.55 weeks of gestation); NPV, negative predictive value; PPV, positive predictive value; Wt, weight.

**Table 4:** Regression analysis of the LBW and SGA neonates on Maternal MUAC1 with different cut‐offs

	LBW			SGA		
Independent variable	OR	*P* value	95% CI	OR	*P* value	95% CI
MUAC <24	3.405	.006	1.43, 8.1	2.74	.009	1.28, 5.84
MUAC <23	1.083	.86	0.46, 2.58	0.902	.79	0.42, 1.93
MUAC <22	1.54	.59	0.47, 3.76	0.97	.95	0.4, 2.34

*Note*. Significant at *P* value ≤ .05.

Abbreviations: LBW, low birthweight; MUAC, mid‐upper arm circumference; OR, odds ratio; SGA, small for gestational age.

Maternal weight and MUAC at all the three time points (20–26 weeks', 30–34 weeks', and >36 weeks' gestation) were available for 485 mother neonate pairs. Table 5 shows Pearson's correlation coefficients between longitudinal measurements of mothers' anthropometry and newborns' anthropometry for 485 mother neonate pairs. Maternal age, height, and weight (three time points), MUAC (three time points), GAD (weeks), and post‐natal weight showed positive correlation with BW, crown heal length, and head circumference of the neonates. Gestational weight gain was also associated with BW (*r* = .202) and length (*r* = .138) but not with head circumference (*r* = −.001).

**Table 5:** Pearson's correlation coefficients (and *P* values) between anthropometric measurements in 485 pregnant woman and their newborn children

	Birthweight (g)		Length (cm)		HC (cm)	
	*r*	*P*	*r*	*p*	*r*	*P*
Age	.119^**^	.009	.154^**^	.002	.153^**^	.002
Ht	.210^**^	.0011	.205^**^	.0011	.186^**^	.001
Wt1	.251^**^	.001	.220^**^	.0011	.196^**^	.001
Wt2	.288^**^	.0011	.253^**^	.0011	.192^**^	.001
Wt3	.316^**^	.0011	.265^**^	.0011	.192^**^	.001
GWG	.202^**^	.0011	.138^**^	.005	−.001	.99
MUAC1	.199^**^	.0011	.162^*^	.001	.123^*^	.014
MUAC2	.213^**^	.0011	.194^**^	.0011	.142^**^	.005
MUAC3	.253^**^	.0011	.191^**^	.0011	.142^**^	.007
PWT	.288^**^	.0011	.220^**^	.0011	.151^**^	.002
GAD	.321^**^	.0011	.154^**^	.002	.146^**^	.003

Abbreviations: GWG, gestational weight gain; HC, head circumference of the neonates; Ht, height; Length, crown heal length; MUAC, mid‐upper arm circumference; PWt, post‐natal weight of women; Wt, weight.

* Correlation is significant at the 0.05 level.

** Correlation is significant at the 0.01 level.

### 3.2 Association of MUAC and skinfolds with maternal weight gain and birth outcome

**Figure 2:**
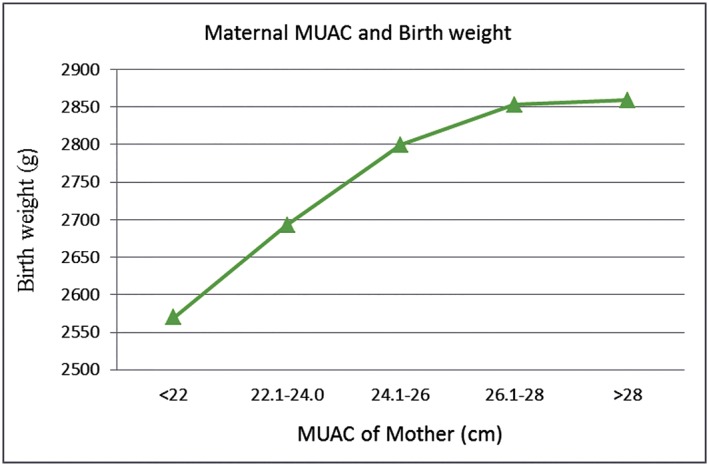
Linear correlation of birthweight with maternal mid‐upper arm circumference (MUAC)

As expected, Wt and MUAC had robust correlations at all the three time points of gestation (*r* = .890, *P* = .001; *r* = .861, *P* = .001; *r* = .844, *P* = .001). Furthermore, Pearson's correlation showed a significant association (*r* = .554; *P* = 000) between MUAC change and gestational weight gain during the course of pregnancy in this cohort. Similarly, there was strong association between MUAC changes and BW. Linear association between maternal MUAC at all three time points during pregnancy (20–24 weeks', 30–34 weeks', and >36 weeks' gestation) with mean BW of the neonates is depicted in Figure 2. As for skinfolds, Pearson correlation showed a significant association of maternal total body fat and lean body mass at all three time points with neonatal anthropometry such as BW, crown heel length, and head circumference (Table S1).

## 4 DISCUSSION

MUAC cut‐off value of ≤23 cm measured during first, second, and third antenatal visits emerged as a good predictor of LBW and SGA neonates in this cohort. Maternal weight and height and as expected gestational age at delivery were related not only to BW but also to crown heel length and head circumference. This is the first study to demonstrate the relationship between MUAC and maternal weight gain on three serial measurements. Pearson's correlation showed association (*r* = .554; *P* = 000) between MUAC changes and gestational weight gain during the 15‐week period of pregnancy in this cohort. Pregnant women with lower height, weight, MUAC, lower fat mass, and lean body mass had smaller babies. The best cut‐off limit with the highest sensitivity–specificity product for weight was 55 kg and height was 152 cm for LBW.

The incidence of LBW and anaemia in the present study was well within the prevalence range reported from India (National Family Health Survey [NFHS‐4], Report 2015‐16: India, 2018). Moreover, the subjects in the current study belong to disadvantaged population and represents majority of women in India. Most studies have shown similar sensitivity and specificity values for MUAC. Predictably, weight also showed similar sensitivity and specificity values to identify women at risk of LBW. In a community‐based longitudinal study conducted by Nahar et al., the sensitivity and specificity values (45 and 59) of maternal weight for predicting LBW were similar to the present study, and the authors stated that maternal weight best predicted the BW. However, obviating pre‐pregnancy weight was their limitation. Though weight was the traditional marker, when presenting late for the antenatal checkups and in resource poor settings, MUAC acts as a simple tool to assess poor pregnancy outcomes (Katz, Khatry, LeClerq, West, & Christian, 2010; Lechtig, 1988; Mohanty et al., 2005; Ricalde et al., 1998; Roy & Sen, 2018).

WHO (Recommendations on antenatal care, 2016) has recommended that MUAC may be useful to identify under nutrition in pregnant women. However, the WHO suggests that the optimal cut‐off points must be determined for individual countries based on context‐specific cost‐benefit analyses. This study helped us to identify a cut‐off value of MUAC 23 cm as risk indicator for LBW and SGA. Its performance was similar to other established anthropometric indicators. In non‐pregnant women, a study by Rodrigues et al. suggested a similar cut‐off value of 24 cm as a sensitive marker for identification of women with BMI<18.5 with sensitivity and specificity of 71.1% percent and 69.6%, respectively. The MUAC value of ≤23 cm is recommended to include pregnant women at risk of LBW for infants in the Asian contexts (Ververs et al., 2013). The WHO Collaborative Study in1997 (Kelly, Kevany, De Onis, & Shah, 1996) also showed MUAC cut‐off values of ≤23 cm as having significant risk for LBW (OR 1.9, 95% CI 95% [1.7, 2.1]). The observations made in the current study indicate an odds of nearly threefold higher risk of having LBW or SGA babies with a MUAC of <24 cm; however, MUAC value of 24 cm had very poor specificity though the sensitivity was higher. Hence, the observations in the current study recommends a MUAC value of ≤23 cm. Mohanty et al. studied 395 singletons, full‐term neonates and suggested a lower MUAC cut‐off (≤22.5 cm) as the best predictor for LBW, but MUAC data were taken from antenatal visit records in the first trimester. Similarly, Sen et al. and Shrivastava et al. suggested a lower MUAC cut‐off of <22 cm and <23 cm, respectively, to be the best surrogate measure of LBW, but these studies collected MUAC measurements at postpartum period and therefore are not comparable with our study. As LBW has detrimental effects on a child's health and survival, a more inclusive approach with a MUAC cut‐off of ≤23 cm should be used to indicate risk of LBW or SGA and to use as entry criterion for nutritional programmes.

There are eight cross‐sectional studies (measured MUAC at the time of labour or postpartum) that analysed maternal MUAC and LBW outcome, of which six studies showed positive association between MUAC and LBW, whereas two studies did not show any correlation (Elshibly & Schmalisch, 2008; Villamor et al., 2002; Ojha & Malla, 2007; Dhar & Bhadra, 2008; Shrivastava et al., 2016; Ricalde et al., 1998; López et al., 2011). Six longitudinal studies that measured MUAC during antenatal visits also reported MUAC as predictor of LBW in pregnant women, similar to the current study. All the six found significantly increased risk of LBW among mothers with low MUAC during pregnancy (Frison et al., 2016; Sebayang et al., 2012; Assefa, Berhane, & Worku, 2012; Mohanty et al., 2005; Kelly et al., 1996; Karim & Mascie‐Taylor, 1997). However, of the six longitudinal studies that identified MUAC as predictor of LBW, two were on HIV population and therefore not comparable with our study. Sebayang et al. studied 14,040 births in Indonesia to examine the determinants of LBW and concluded that MUAC < 23.5 cm or short stature (height < 145 cm), or both increased the likelihood of having a LBW baby.

In addition to LBW, we also compared BW with fetal growth standards by week of gestation and identified 33% neonates to be SGA for both preterm and full‐term births. SGA is a surrogate marker of fetal growth restriction. There are myriad causes of fetal growth restriction, but as observed in the current study, maternal weight, BMI, and weight gain during pregnancy are strong indicators predicting LBW and SGA. One study by Sebayang et al. reported MUAC as a predictor of SGA similar to our findings.

Limitations of the study include usage of right arm and lack of data on MUAC and maternal weight during the first trimester, which would have added more insight on the outcome. Major strengths of the study include serial measurements of maternal MUAC at three time points during antenatal period by well‐trained field staff; and this is the first prospective study that measured MUAC during the course of pregnancy in the same cohort. Unlike many other researches, data on BW were collected within 24 hr after delivery.

Although recognized earlier, the importance of MUAC in predicting BW was first published by Lechtig (1988) in a study comparing MUAC and other conventional high‐risk anthropometric indicators during pregnancy for LBW assessment in Guatemala. Being simple and cost‐effective, MUAC was rapidly promoted as an indicator for risk of LBW baby by many longitudinal and cross‐sectional studies (Dhar & Bhadra, 2008; Elshibly & Schmalisch, 2008; Sebayang et al., 2012). LBW or SGA babies are not only at greater risk of dying than infants of average weight but also at risk of more frequent infections and impaired cognitive development and are more likely to become undernourished children and adolescents (Saugstad, 1981). Evidence is now pointing that LBW/SGA predisposes children to a high risk of diabetes, heart diseases, and other chronic conditions later in life (Barker, 1990). Hence, it is an urgent need to identify pregnant women at risk, to decrease the burden of LBW or SGA babies. MUAC is a simple, easy to conduct marker of maternal nutrition status and therefore has been suggested to identify women at risk of delivering LBW.

BW less than 2.5 kg, defined as LBW, is a poor outcome as a consequence of being born prematurely, having fetal growth restriction or both. Globally, an estimated 20 million births a year are LBW, which is about 20% of all live births, but most of the LBW babies are born in developing countries, and India contributes to 30% of global LBWs (WHO‐GNT 2025:2015). There are ongoing nutrition supplementation programmes for adolescent girls and pregnant and lactating women in India. Identifying additional women as high risk based on MUAC might lead to additional allowance of food supplements especially protein‐rich foods such as milk and eggs, apart from care and increased follow‐up visits for counselling.

Knowledge about MUAC changes during the course of pregnancy and the cut‐off that could predict LBW is limited (Frison et al., 2016). MUAC of <22 cm has been suggested as an indicator of wasting, but MUAC cut‐off points to predict LBW are suggested to be different for different regions (Villamor et al., 2002). Moreover, MUAC collected during any point of time during pregnancy has been proposed to be able to predict LBW.

## 5 CONCLUSION

The current study demonstrated predictive ability of MUAC ≤23 cm at all three time points during pregnancy (20–24 weeks', 30–34 weeks', and >36 weeks' gestation). Maternal MUAC cut‐off of ≤23 cm from 20 weeks' gestation up till delivery can be considered for prediction of LBW. However, further studies need to be taken up with a nationally representative sample to validate this method.

## CONFLICTS OF INTEREST

The authors declare that they have no conflicts of interest.

## CONTRIBUTIONS

RH designed the study concept and substantially contributed in executing and writing the manuscript. VD and RB handled the data collection. BV and RS substantially contributed in data analysis. RK, SS, and JJ involved in interpreting the results. RH and VD wrote the manuscript and all the authors reviewed and approved the final manuscript.

## Supporting information

Table S1. Maternal body composition at different stages of gestation with birth weight, length and HC
