# Supplementary material for: Maternal MUAC and fetal outcome in an Indian tertiary care hospital: A prospective observational study
Source: Matern Child Nutr. 2019 Dec 12;16(2):e12902. doi: 10.1111/mcn.12902 (PMC7083480; doi:10.1111/mcn.12902)

**Maternal Mid-Upper Arm Circumference and foetal outcome in a South Indian tertiary care hospital: a prospective observational study**

**Short title:** Maternal MUAC and foetal outcome

**Abstract**

Studies to date demonstrated the relatedness of mid-upper arm circumference (MUAC) measurement of pregnant women to their anthropometry/weight. Hence the objective was to determine whether maternal MUAC at different gestational age predicted birth weight, and if so, to identify which cut-offs provided the best prediction of low birth weight (LBW) in pregnant women cohort. A total of 928 pregnant women, free of any obstetrical and medical complications known to affect foetal growth were followed from 20-24 weeks gestation till delivery. Weight, height and MUAC were determined for the pregnant women, and gestational age along with newborns anthropometry was collected. The mean birth weight was 2.6 ± 0.460 kg. Maternal age, height, weight, MUAC (three time points), Gestational age at delivery and postnatal weight showed positive correlation with birth weight, crown heel length and head circumference of the neonates. The cut-off limit with the best sensitivity-specificity (54.0 and 59.8 respectively) for MUAC was 23 cm, while maternal weight of 55kg had sensitivity and specificity of 62.5 and 59.9 for predicting LBW. Maternal weight of 55 kg and MUAC value of 23 cm had almost similar sensitivity and specificity for predicting LBW. MUAC (≤ 23cms) can be considered as a potential indicator of LBW where weighing of pregnant women is not feasible or when presentation for antenatal care is late, especially where pre-pregnancy weights are not available.

**Key words:** Birth weight, Foetal outcome, Gestational weight gain, Low birth weight, MUAC, Pregnancy, SGA

**1 Introduction**

Low birth weight is recognised as an important determinant of neonatal mortality and morbidity and it is one of the World Health Assembly targets by 2025. The annual average rate of reduction in low birth weight prevalence globally was only 1.2% between the year 2000 and 2015 (UNICEF-WHO Low birth weight estimates, 2019). To affect the future survival of the newborn and to improve quality of adult life, neonatal birth weight should be improved, which in turn is directly dependent on maternal anthropometry (Sen et al., 2010). Considerable attention has been focused on the maternal anthropometric measurements as indicators of low birth weight for identifying women at risk of LBW. Impaired nutritional status of women before conception, short stature and poor nutrition during pregnancy are important contributing factors of low birth weight (Sen et al., 2010; Muthayya et al., 2009; WHO- Provisional agenda item 6.3, 2011).

Maternal anthropometry such as maternal weight (Wt), height (Ht), mid-upper circumference (MUAC), and maternal body mass index (BMI) in the first trimester are suggested as good predictors of LBW. Nevertheless, pre-pregnancy body mass index and gestational weight gain are the preferred anthropometric indicators to identify women at risk of producing LBW babies. However, in India, pregnant women begin to visit antenatal clinics after 10 to 12 weeks of pregnancy and therefore pre-pregnancy weight may not be available to calculate BMI. Considering various influencing factors, studies have demonstrated that MUAC is closely related to maternal weight and therefore it has been suggested as an effective tool for maternal nutrition status screening (Elshibly et al., 2008; Tang et al., 2016; Lechtig et al., 1988). However, knowledge about its changes during the course of pregnancy and the cut off that could predict LBW is limited (Frison et al., 2016). There is lacuna of established cut-off points of maternal MUAC by taking demographic differences into account. MUAC of <22cm has been suggested as an indicator of wasting, but MUAC cut-off points to predict LBW are suggested to be different for different regions (Villamor et al., 2002). Yet most studies have measured MUAC just before or after delivery; data on changes in MUAC values during pregnancy are limited (Tang et al., 2016; Ojha et al., 2007; Dhar et al., 2008; Sebayang et al., 2012; Aseefa et al., 2012; Shrivastava et al., 2016; Mohanty et al., 2006; Recalde et al., 1998; Lopez et al., 2011). Recalde *et al.* collected three serial measurements on 92 pregnant women in Brazil and Lopez *et al.* determined pattern of changes in mid-upper arm circumference, triceps, biceps and subscapular skinfold thicknesses during the course of pregnancy. In 2016, WHO has recommended that MUAC may be useful to identify undernourished pregnant women and suggested that the optimal cut-off points must be determined for individual countries based on context-specific cost–benefit analyses (World Health Organization-Recommendations on antenatal care, 2016). Keeping in view the above, the current prospective cohort study determined maternal MUAC at different gestational age and also assessed the sensitivity of predicting women at risk of LBW.

Key messages

- Though MUAC is identified as a marker to assess nutritional status of pregnant women by WHO, data on optimal cut-off points is lacking especially in Indian context.
- This is the first study to demonstrate relationship between MUAC and maternal weight gain on three serial measurements.
- We found a similar sensitivity-specificity of MUAC for predicting LBW to that of maternal weight; suggesting the validation of MUAC.
- Contributing to validate cut-off point of MUAC measurements to assess poor pregnancy out-comes, which can help in identifying women at risk.

**2 Methods**

**2.1 Study Setting**

In a prospective cohort study, pregnant women who received antenatal care at a tertiary care government maternity hospital located at Hyderabad, Telangana and were delivered at the same hospital from the period, May 2012 to May 2015, were screened.

**2.2 Sample size calculation**

Assuming a sensitivity of 60% with a similar specificity of 60%, taking a precision of 10% and 95% Confidence Interval with 80% power the sample size required was 556. However, expecting attrition of 35% due to long follow-up, the sample size was calculated to 751. But 928 were found to be eligible and were recruited for screening (Nahar et al., 2007; Roy et al., 2018).

**2.3 Participants**

Pregnant women in their first and second trimester, willing to participate in the study were included for the recruitment. Women with gestational age nearing 30 weeks, gestational diabetes, severe anaemia, pre-eclampsia, chronic hypertension, foetal anomaly, rheumatoid arthritis, thyroid and parathyroid disorders, hepatic or renal or cardiovascular diseases were excluded from the study. Nine hundred and twenty eight (928) pregnant women, who fulfilled inclusion criteria were registered after obtaining written informed consent and were followed through during pregnancy till child birth. Of the 928, 615 and 563 turned up for follow up during 30-34 weeks and >36 weeks gestation respectively; but delivery data such as birth weight and gestational age were collected from 804 of 928 recruited women. Only singleton deliveries were included in the final analysis of the data. In India various programs like ‘Janani Suraksha Yojana’ under the National Rural Health Mission are functional under which women are paid a substantial fund for each ANC (ante-natal check) visit to encourage ANCs and Institutional deliveries. This enabled us to get good follow-up of the study cohort. In the state where the study was conducted, 74.9% of pregnant women had 4 antenatal care visits to the health centers (Fact sheets-2015-16, NHFS-4, India). All procedures performed in the study were in accordance with the ethical standards of the institutional and/or national research committee and with the 1964 Helsinki declaration and its later amendments.

**2.4 Procedures**

Anthropometric indicators include maternal Wt in kg, Ht in cm and MUAC in cm. BMI was calculated by taking pregnant women’s weight in kg divided by her height in meters squared. BMI of less than 18.5 was classified as chronic energy deficiency or undernourished. Wt, MUAC and skin folds at four sites were collected at three time points during pregnancy (20-26 weeks, 30-34 weeks and >36 weeks gestation). MUAC was measured in the right arm at the level, mid way between acromion and olecranon processes in cm, to the nearest decimal place. Triceps, biceps and subscapular skinfold thickness was measured by trained nutritionists using a Lange skinfold caliper (nearest to 1mm) according to standardized methods. The average of three measurements was recorded at each site. Maternal body composition was evaluated for all the three times during gestation. Postnatal weight was collected after 24 hours, but within 5 days after delivery. Two dedicated project staff, nutritionists were doing the anthropometry after due training and inter / intra rater reliability was done every 3 months to keep the CV below 10%. All the demographic and pregnancy details along with 24hour diet recall were collected from the participants.

The babies were examined within 24 h of delivery and birth weights, crown heel length and head circumference were recorded using the Seca weighing scales (to the nearest 1g), infanto-meter and measuring tape (to the nearest 1cm) respectively. As per the WHO (1995) definition, newborns weighing less than 2.5kg were considered as LBW neonates. Gestational age was determined by dating the last menstrual period and at the time of recruitment and was corrected by first trimester ultrasonographic findings if the difference exceeded 5 days. SGA neonates were determined by comparing the birth weight with foetal growth standard by gestation week. Neonates with birth weight less than 10^th^ percentile of the standard population for gestation week were considered as small for gestational age (Papageorghiou AT et al., 2014). SGA is a surrogate marker for identification of newborns with foetal growth restriction.

**Flow Chart of the study**

1220 pregnant women (PW) screened at Government Maternity Hospital, Hyderabad (May 2012-May 2015)

292 PW excluded for various obstetrical complications and under treatment

928 PW fulfilled the eligibility criteria

313 PW did not turn-up for 1^st^ follow up

615 PW turned up for follow up at 30-34 weeks of gestation

52 PW did not turn –up for 2^nd^ follow up

563 came for 2^nd^ follow up visit at >36 weeks of gestation

124 lost to follow-up and of 928 registered PW, 804 came for delivery in the same hospital.

Birth weight and gestational age data collected for 804 study participants (out of 928 cases)

Data available for analysis:

1. 563 participants with 2 follow up visits
2. 804 participants Birth weight and gestational age

**2.5 Statistical Analysis**

Data was analyzed using SPSS 17.0 for windows IBM, Chicago. Mean and SD were calculated, mothers’ Wt, Ht, MUAC and BMI were ascertained for a birth weight of 2.5 kg. Odds ratios were computed to assess the risk of LBW between various cut off points of MUAC and 95 percent confidence intervals were calculated. Adjusted odds ratios from multivariable regression models were also performed. In addition, sensitivity, specificity, positive predictive value, negative predictive value, and area under the receiver operating characteristic curve (AUC) were calculated for predicting birth weight outcome for various MUAC cut offs. Pearson’s Correlation coefficients between longitudinal measurements of mothers’ anthropometry and newborns’ anthropometry was done. Significance considered at p.value 0.05.

**3 Results**

A total of 928 pregnant women were followed from 20-24 weeks gestation till delivery. Mean age of the pregnant women was 21.9 ± 2.642 and 1.4% were less than 18 years age. 17.8% of them were illiterate and 14.6% were moderate workers the rest being sedentary. However, all the study participants belonged to low socio-economic status based on Kuppuswamy’s guidelines (Bairwa M et al., 2016). Mean energy (1980±829 kcals) and fat (33.8±7.2gms) intakes were within recommended levels, while protein intake (54.66±29.8gms) was lower than the recommended values of RDA for pregnant women. All the study participants were non-smokers, non-alcoholic and free of any obstetrical and medical complications known to affect fetal growth.The mean±SD gestational age at the time of recruitment was 23.6±6.55. Of the 928 pregnant women followed, birth outcome data were collected from 804 pregnant women. Of the 804, 50.7% babies were males and the rest were females, and 73% delivered normally while 27% had caesarian section. The newborns were free of any congenital anomalies. The mean birth weight was 2.6 ± 0.460 kg. Frequency of LBW and pre term births (PTB) were 22.1% and 7.1 % respectively and 33% were SGA. One third (33%) of the babies were less than 10^th^ percentile of standard population ([Papageorghiou AT](https://www.ncbi.nlm.nih.gov/pubmed/?term=Papageorghiou AT%5BAuthor%5D&cauthor=true&cauthor_uid=25209488) et al., 2014) and were classified as SGA.

Maternal weight of the cohort ranged from 32 to 91 kg, and merely 19.2 % were weighing more than 55 kg and 44.6% were between 45 to 55 kg at the time of recruitment. A proportion of 36.2 % women in the study were weighing less than 45kg. MUAC ranged from 16 to 35.5 cm; and 27.3 % were wasted when MUAC of less than 22 cm was considered. Ht showed that 64.7 % had greater than 150 cm and a proportion of 52.7 % had height greater than 152cm. As for haemoglobin (Hb) status, only 23.2 % had Hb concentration more than 11 g/dl. While 55.3 % had mild anemia (9 to 11g/dl), 18.4 % had moderate anemia (7 to 9 g/dl) and severe anemia was prevalent in 3.2% of pregnant women at the time of recruitment.

Mean values of Wt and MUAC collected serially during 3 time points (20-24 weeks, 30-34 weeks and >36 weeks gestation) are given in Table 1. The mean height was 152.1 ± 5.606 cm and the mean±SD Wt was 48.6 ± 8.128 kg at the time of recruitment, which increased to 56.2 ± 8.788 kg by 38.5±2.09 weeks of gestation. Over the same period, mean±SD maternal MUAC increased from 23.7 ± 2.863 cm to 24.5 ± 2.866 cm. Predictably, mean age, Ht, Wt, MUAC and gestational age at delivery (GAD) were significantly low in LBW (Table 1). Post-natal maternal weight was also low in LBW (Table 1). Likewise, Wt and MUAC during 3 time points were significantly low in SGA neonates (Table 2). Mean values of MUAC of pregnant women at 23 weeks gestation was 23.7 cm (±2.86), at 32 weeks 24.5cm (±2.94) and at 38 weeks of gestation the MUAC was 24.5 cm (±2.86). MUAC was similar at different time points during pregnancy.

**3.1 Mothers’ Wt, Ht and MUAC values corresponding to birth weight**

Table 3 shows the serial cut- off values and validity indices of Wt, Ht and MUAC as an indicator of LBW. The best cut-off limit with the highest sensitivity-specificity product for Wt was 55 kg and Ht was 152cm. Similarly, MUAC value of 23 cm had sensitivity and specificity of 54.0 and 59.8, while at 24 cm the sensitivity rose to 71.3, but specificity decreased with an AUC value of 0.57. The mothers’ Wt, Ht and MUAC values corresponding to birth weight (BW) of 2500 g was calculated using the regression equation. The regression equation, 35.562+5.027 BW considering the mothers’ Wt corresponding to a BW of 2500 g was calculated to be 48.1kg. Ht was calculated to be 152.1cm using the regression equation, 145.229+2.732 BW. Similarly, the maternal MUAC corresponding to a birth weight of 2500 g was calculated to be 23.66 cm using the regression equation, 20.236+1.372 BW. Regression analysis of the LBW and the SGA neonates on Maternal MUAC1 with different cutoffs showed 3.4 folds and 2.7 folds higher odds of having LBW or SGA neonates if the MUAC was less than 24 cm (Table 4).

Maternal weight and MUAC at all the three time points (20-26 weeks, 30-34 weeks and >36 weeks gestation) were available for 485 mother neonate pairs. Table 5 shows Pearson’s Correlation coefficients between longitudinal measurements of mothers’ anthropometry and newborns’ anthropometry for 485 mother neonate pairs. Maternal age, Ht and Wt (three time points), MUAC (three time points), GAD (weeks) and Postnatal weight showed positive correlation with birth weight, crown heal length and head circumference of the neonates. Gestational weight gain was also associated with birth weight (r=0.202) and length(r=0.138), but not with head circumference (r=-0.001).

**3.2 Association of MUAC and skin folds with maternal weight gain and birth outcome**

As expected, Wt and MUAC had robust correlations at all the three time points of gestation (r=0.890, p=0.001; r=0.861, p=0.001; r=0.844, p=0.001). Furthermore, Pearson’s correlation showed a significant association (r=0.554; p=000) between MUAC change and Gestational weight gain during the course of pregnancy in this cohort. Similarly, there was strong association between MUAC changes and birth weight. Linear association between maternal MUAC at all 3 time points during pregnancy (20-24 weeks, 30-34 weeks and >36 weeks gestation) with mean birth weight of the neonates is depicted in the figure 1. As for skin folds, Pearson correlation showed a significant association of maternal total body fat and lean body mass at all three time points with neonatal anthropometry such as birth weight, crown heel length and head circumference (supplementary Table 1).

**4 Discussion**

MUAC cutoff value of ≤ 23 cm measured during first, second and third antenatal visits emerged as a good predictor of LBW and SGA neonates in this cohort. Maternal weight and height, and as expected gestational age at delivery were related not only to birth weight, but also to crown heel length and head circumference. This is the first study to demonstrate the relationship between MUAC and maternal weight gain on three serial measurements. Pearson’s correlation showed association (r=0.554; p=000) between MUAC changes and Gestational weight gain during the 15 week period of pregnancy in this cohort. Pregnant women with lower height, weight, MUAC, lower fat mass and lean body mass had smaller babies. The best cut-off limit with the highest sensitivity-specificity product for weight was 55 kg and height was 152cm for LBW.

The incidence of LBW and anemia in the present study was well within the prevalence range reported from India (National Family Health Survey (NFHS-4), Report 2015-16: India). Moreover, the subjects in the current study belong to disadvantaged population and represents majority of women in India. Most studies have shown similar sensitivity and specificity values for MUAC. Predictably, Wt also showed similar sensitivity and specificity values to identify women at risk of LBW. In a community based longitudinal study conducted by S Nahar et al., the sensitivity and specificity values (45 and 59) of maternal weight for predicting LBW were similar to the present study, and the authors stated that maternal weight best predicted the birth weight. However, obviating pre-pregnancy weight was their limitation. Though Wt was the traditional marker, when presenting late for the antenatal checkups and in resource poor settings, MUAC acts as a simple tool to assess poor pregnancy out-comes (Lechtig et al., 1988; Mohanty et al., 2006; Ricalde et al., 1998; Roy et al., 2018; Katz et al., 2010).

In 2016, the WHO has recommended that MUAC may be useful to identify under nutrition in pregnant women. However, the WHO suggests that the optimal cut-off points must be determined for individual countries based on context-specific cost–benefit analyses (WHO-Recommendations on antenatal care, 2016). This study helped us to identify a cut off value of MUAC 23 cm as risk indicator for LBW and SGA. Its performance was similar to other established anthropometric indicators. In non-pregnant women, a study by Rodrigues *et al.* suggested a similar cut off value of 24 cm as a sensitive marker for identification of women with BMI<18.5 with sensitivity and specificity of 71.1 percent and 69.6 percent respectively. The MUAC value of ≤ 23 cm is recommended to include pregnant women at risk of LBW for infants in the Asian contexts (Verves et al., 2013). The WHO Collaborative Study in1997 (Kelly et al., 1997) also showed MUAC cut-off values of ≤ 23 cm as having significant risk for LBW (OR 1.9, 95% CI: 95% 1.7-2.1). The observations made in the current study indicate an odds of nearly 3 fold higher risk of having LBW or SGA babies with a MUAC of <24 cm; however, MUAC value of 24 cm had very poor specificity though the sensitivity was higher. Hence, the observations in the current study recommends a MUAC value of ≤23 cm. Mohanty *et al*. studied 395 singletons, full term neonates and suggested a lower MUAC cutoff (≤22.5 cm) as the best predictor for low birth weight, but MUAC data was taken from antenatal visit records in the first trimester. Similarly, Sen *et al.* and Shrivastava *et al.* suggested a lower MUAC cutoff of <22cm and <23 cm respectively to be the best surrogate measure of LBW, but these studies collected MUAC measurements at postpartum period and therefore are not comparable to our study. As LBW has detrimental effects on a child’s health and survival, a more inclusive approach with a MUAC cut-off of ≤23 cm should be used to indicate risk of LBW or SGA and to use as entry criterion for nutritional programs.

There are eight cross sectional studies (measured MUAC at the time of labor or postpartum) that analyzed maternal MUAC and LBW outcome, of which six studies showed positive association between MUAC and LBW, while two studies did not show any correlation (Elshibly et al., 2008; Villamor et al., 2002; Ojha et al., 2007; Dhar et al., 2008; Shrivastava et al., 2016; Ricalde et al., 1998; Lopez et al., 2011). Six longitudinal studies that measured MUAC during antenatal visits also reported MUAC as predictor of LBW in pregnant women, similar to the current study. All the six found significantly increased risk of LBW among mothers with low MUAC during pregnancy (Frison et al., 2016; Sebayang et al., 2012; Assefa et al., 2012; Mohanty et al., 2006; Kelly et al., 1997; Karim et al., 1997). However, of the six longitudinal studies that identified MUAC as predictor of LBW, two were on HIV population and therefore not comparable with our study. Sebayang *et al.* studied 14,040 births in Indonesia to examine the determinants of low birth weight and concluded that MUAC < 23.5 cm or short stature (height < 145 cm), or both increased the likelihood of having a LBW baby.

In addition to LBW, we also compared birth weight with foetal growth standards by week of gestation and identified 33 percent neonates to be SGA for both preterm and full-term births. SGA is a surrogate marker of foetal growth restriction. There are myriad causes of foetal growth restriction, but as observed in the current study, maternal weight, BMI and weight gain during pregnancy are strong indicators predicting LBW and SGA. One study by Sebayang *et al.* reported MUAC as a predictor of SGA similar to our findings.

Limitations of the study include usage of right arm and lack of data on MUAC and maternal weight during the first trimester, which would have added more insight on the outcome. Major strengths of the study include serial measurements of maternal MUAC at three time points during antenatal period by well trained field staff; and this is the first prospective study that measured MUAC during the course of pregnancy in the same cohort. Unlike many other researches, data on birth weight were collected within 24 hours after delivery.

Although recognised earlier, importance of MUAC in predicting birth weight was first published by Lechtig A in a study comparing MUAC and other conventional high-risk anthropometric indicators during pregnancy for LBW assessment in Guatemala. Being simple and cost effective, MUAC was rapidly promoted as an indicator for risk of LBW baby by many longitudinal and cross sectional studies (Elshibly et al., 2008; Dhar et al., 2008; Sebayang et al., 2012). LBW or SGA babies are not only at greater risk of dying than infants of average weight, but they are at risk of more frequent infections, impaired cognitive development and are more likely to become undernourished children and adolescents (Saugstad et al.,1981). Evidence is now pointing that LBW/ SGA predisposes children to a high risk of diabetes, heart diseases and other chronic conditions later in life (Barker et al., 1990). Hence it is an urgent need to identify pregnant women at risk, to decrease the burden of LBW or SGA babies. MUAC is a simple, easy to conduct marker of maternal nutrition status and therefore has been suggested to identify women at risk of delivering LBW.

Birth weight less than 2.5kg, defined as LBW is a poor outcome as a consequence of being born prematurely, having foetal growth restriction or both . Globally, an estimated 20 million births a year are LBW, which is about 20 percent of all live births, but most of the LBW babies are born in developing countries and India contributes to 30% of global LBWs (WHO-GNT 2025:2015). There are ongoing nutrition supplementation programs for adolescent girls and pregnant & lactating women in India. Identifying additional women as high risk based on MUAC, might lead to additional allowance of food supplements especially protein rich foods such as milk, eggs etc., apart from care and increased follow up visits for counseling etc.

Knowledge about MUAC changes during the course of pregnancy and the cut off that could predict LBW is limited (Frison et al., 2016). MUAC of <22cm has been suggested as an indicator of wasting, but MUAC cut-off points to predict LBW are suggested to be different for different regions (Villamore et al., 2002). Moreover, MUAC collected during any point of time during pregnancy has been proposed to be able to predict LBW.

**5 Conclusion**

The current study demonstrated predictive ability of MUAC ≤23 cm at all 3 time points during pregnancy (20-24 weeks, 30-34 weeks and >36 weeks gestation). Maternal MUAC cut off of ≤23 cm from 20 weeks gestation up till delivery can be considered for prediction of low birth weight. However, further studies need to be taken up with a nationally representative sample to validate this method.

**References**

1. Assefa, N., Berhane, Y., & Worku, A. (2012). Wealth status, mid upper arm circumference (MUAC) and antenatal care (ANC) are determinants for low birth weight in Kersa, Ethiopia. *PloS one*, *7*(6), e39957.
2. Bairwa, M., Rajput, M., & Sachdeva, S. (2013). Modified Kuppuswamy’s socioeconomic scale: social researcher should include updated income criteria, 2012. *Indian journal of community medicine: official publication of Indian Association of Preventive & Social Medicine*, *38*(3), 185.
3. Barker, D. J. (1990). The fetal and infant origins of adult disease. *BMJ: British Medical Journal*, *301*(6761), 1111.
4. Dhar, B., & Bhadra, S. K. (2008). Use of anthropometric indicators for predicting risk of delivering low birth weight babies. *Bangladesh Medical Research Council Bulletin*, *34*(2), 64-66.
5. Elshibly, E. M., & Schmalisch, G. (2008). The effect of maternal anthropometric characteristics and social factors on gestational age and birth weight in Sudanese newborn infants. *BMC public health*, *8*(1), 244.
6. Frison, S., Kerac, M., Checchi, F., & Prudhon, C. (2016). Anthropometric indices and measures to assess change in the nutritional status of a population: a systematic literature review. *BMC nutrition*, *2*(1), 76.
7. Habicht, J. P., & Yarbrough, C. H. A. R. L. E. S. (1979). Efficiency in selecting pregnant women for food supplementation during pregnancy.
8. Karim, E., & Mascie-Taylor, C. G. N. (1997). The association between birthweight, sociodemographic variables and maternal anthropometry in an urban sample from Dhaka, Bangladesh. *Annals of human biology*, *24*(5), 387-401.
9. Katz, J., Khatry, S. K., LeClerq, S. C., West, K. P., & Christian, P. (2010). The post‐partum mid‐upper arm circumference of adolescents is reduced by pregnancy in rural Nepal. *Maternal & child nutrition*, *6*(3), 287-295.
10. Kelly, A., Kevany, J., De Onis, M., & Shah, P. M. (1996). A WHO collaborative study of maternal anthropometry and pregnancy outcomes. *International Journal of Gynecology & Obstetrics*, *53*(3), 219-233.
11. Lechtig, A. (1988). Predicting risk of delivering low birthweight babies: which indicator is better?. *Journal of tropical pediatrics*, *34*(1), 34-41.
12. López, L. B., Calvo, E. B., Poy, M. S., del Valle Balmaceda, Y., & Cámera, K. (2011). Changes in skinfolds and mid‐upper arm circumference during pregnancy in Argentine women. *Maternal & child nutrition*, *7*(3), 253-262.
13. Mavalankar, D. V., Trivedi, C. C., & Gray, R. H. (1994). Maternal weight, height and risk of poor pregnancy outcome in Ahmedabad, India. *Indian pediatrics*, *31*(10), 1205-12.
14. Mohanty, C., Prasad, A. R., Reddy, S., Jayant, K. G., Singh, T. B., & Das, BK. (2005). Maternal Anthropometry as Predictors of Low Birth Weight. Journal of Tropical Pediatrics. 52(1), 24–29.
15. Muthayya, S. (2009). Maternal nutrition & low birth weight-what is really important. *Indian J Med Res*, *130*(5), 600-8.
16. Nahar, S., Mascie-Taylor, C. G. N., & Begum, H. A. (2007). Maternal anthropometry as a predictor of birth weight. *Public health nutrition*, *10*(9), 965-970.
17. National Family Health Survey (NFHS-4), Report 2015-16: India (2018). Retrieved from <http://rchiips.org/NFHS/NFHS-4Reports/India.pdf>
18. Ojha, N., & Malla, D. S. (2007). Low birth weight at term: relationship with maternal anthropometry. *JNMA; journal of the Nepal Medical Association*, *46*(166), 52-56.
19. Papageorghiou, A. T., Kennedy, S. H., Salomon, L. J., Ohuma, E. O., Cheikh Ismail, L., Barros, F. C., ... & Gravett, M. G. (2014). International standards for early fetal size and pregnancy dating based on ultrasound measurement of crown–rump length in the first trimester of pregnancy. *Ultrasound in Obstetrics & Gynecology*, *44*(6), 641-648.
20. Ricalde, A. E., Velásquez-Meléndez, G., Tanaka, A. C. D. A., & de Siqueira, A. A. (1998). Mid-upper arm circumference in pregnant women and its relation to birth weight. *Revista de saude publica*, *32*, 112-117.
21. Rodrigues, VC., Rao, RS., & Lena, A. (1994). Utility of Arm Circumference as a Screening Instrument to Identify Women at Nutritional Risk. *Tropical Doctor*. 24(4), 164–6.
22. Roy, S., & Sen, J. (2018). Review on studies of mid-upper arm circumference among pregnant women. *Human Biology*. 7(3), 280-306.
23. Saugstad L. F. (1981). Weight of all births and infant mortality. *Journal of epidemiology and community health*, *35*(3), 185–191. doi:10.1136/jech.35.3.185.
24. Sebayang, S. K., Dibley, M. J., Kelly, P. J., Shankar, A. V., Shankar, A. H., & SUMMIT Study Group. (2012). Determinants of low birthweight, small‐for‐gestational‐age and preterm birth in Lombok, Indonesia: analyses of the birthweight cohort of the SUMMIT trial. *Tropical Medicine & International Health*, *17*(8), 938-950.
25. Sen, J., Roy, A., & Mondal, N. (2009). Association of maternal nutritional status, body composition and socio-economic variables with low birth weight in India. *Journal of tropical pediatrics*, *56*(4), 254-259.
26. Shrivastava, J., Agrawal, A., & Giri, A. (2016). Maternal anthropometry in relation to birth weight of newborn: A prospective hospital based study. *Indian Journal of Child Health*, *3*(1), 59-63.
27. Tang, A. M., Chung, M., Dong, K., Terrin, N., Edmonds, A., & Assefa, N. (2016). Determining a global midupper arm circumference cutoff to assess malnutrition in pregnant women. Washington (DC): FHI 360. *Food and Nutrition Technical Assistance III Project (FANTA)*.
28. Ververs, M. T., Antierens, A., Sackl, A., Staderini, N., & Captier, V. (2013). Which anthropometric indicators identify a pregnant woman as acutely malnourished and predict adverse birth outcomes in the humanitarian context?. *PLoS currents*, *5*.
29. Villamor, E., Msamanga, G., Spiegelman, D., Coley, J., Hunter, D. J., Peterson, K. E., & Fawzi, W. W. (2002). HIV status and sociodemographic correlates of maternal body size and wasting during pregnancy. *European Journal of Clinical Nutrition*, *56*(5), 415.
30. WHO Global Nutrition Targets 2025: Low birth weight policy brief 3 (2015). Retrieved from <https://www.who.int/nutrition/publications/globaltargets2025_policybrief_lbw/en/>
31. UNICEF-WHO Low birthweight estimates (2019). Retrieved from <https://data.unicef.org/topic/nutrition/low-birthweight/>
32. WHO recommendations on antenatal care for a positive pregnancy experience: evidencebase (2016). Retrieved from <https://www.who.int/reproductivehealth/publications/maternal_perinatal_health/anc-positive-pregnancy-experience/en/>
33. World Health Organization (EB130/11 130th session), 2011. Nutrition of women in the preconception period, during pregnancy and the breastfeeding period. Retrieved from http://apps.who.int/gb/ebwha/pdf_files/EB130/B130_11-en.pdf.

**Figure Legends**

Figure Title: Figure 1. Linear correlation of Birth eight with Maternal MUAC

**Table 1** Maternal and New born anthropometry

|  | Total | Normal  birth weight | Low birth weight | P value |
| --- | --- | --- | --- | --- |
| **Maternal parameters** | | | | |
| Age  (804) | 21.9 ± 2.642 | 22.0 ± 2.614 (626) | 21.5 ± 2.484 (178) | .018 |
| Height (cms) (804) | 152.1 ± 5.606 | 152.6 ± 5.633 (626) | 151.0 ± 5.255 (178) | .001 |
| Maternal  weight in kgs at  23.6±6.55 weeks of gestation (804) | 48.6 ± 8.128 | 49.7 ± 8.290  (626) | 46.5 ± 8.260 (178) | .001 |
| Maternal weight in kgs  at  32.6±3.78 weeks of gestation (615) | 54.1 ± 8.554 | 55.2 ± 8.408   (482) | 51.6 ± 9.042 (133) | .001 |
| Maternal  weight in kgs at  38.5±2.09 weeks of gestation (563) | 56.2 ± 8.788 | 57.3 ± 8.715  (461) | 52.5 ± 8.576 (102) | .001 |
| MUAC  (cms) at  23.6±6.55 weeks of gestation (748) | 23.7 ± 2.863 | 24.0 ±  2.916 (584) | 23.2 ± 2.932 (164) | .001 |
| MUAC  (cms) at  32.6±3.78 weeks of gestation (590) | 24.5 ± 2.941 | 24.8 ± 2.926 (466) | 23.7 ± 3.115 (124) | .001 |
| MUAC (cms) at  38.5±2.09 weeks of gestation (504) | 24.5 ± 2.866 | 24.8 ±2.825  (415) | 23.3 ± 2.861 (89) | .001 |
| HB (gm/dl)  (629) | 9.9 ± 1.447 | 9.9 ± 1.379   (492) | 9.7 ± 1.571 (137) | .321 |
| Gestational age at delivery (weeks) (748) | 38.5 ± 2.090 | 38.9 ± 1.113  (624) | 37.5 ± 2.475 (176) | .001 |
| Post-natal weight  (kgs)   (611) | 50.0 ± 8.796 | 50.6 ± 8.387  (482) | 46.9 ± 8.444 (125) | .001 |
| **New born  parameters** | | | | |
| Birth weight (kgs) | 2.6 ± .460 | 2.8 ± 0.315 (626) | 2.04 ± 0.310 (178) | .001 |
| Length  (cms) | 47.9 ± 2.221 | 48.3 ± 1.994  (463) | 46.0 ± 2.099 (114) | .001 |
| HC (cms) | 32.7 ± 1.286 | 32.9 ± 1.163  (460) | 31.7 ± 1.325 (109) | .001 |

Note. Values are Mean ± Std. Deviation; Figure in parenthesis indicates number of pregnant women; MUAC-Mid Upper Arm Circumference; HB-Haemoglobin; HC-Head Circumference.

**Table 2** Mean and SD of maternal weight and MUAC during different stages of gestation in women with SGA babies

|  | Normal(≥10^th^ percentile) | SGA (<10^th^ percentile) | P - value |
| --- | --- | --- | --- |
| Wt1, (804) | 50.5±8.33 | 46.6±8.11 | 0.001 |
| Wt2 , (615) | 55.9±8.42 | 51.4±7.93 | 0.001 |
| Wt3 , (563) | 58.5±8.57 | 53.5±8.20 | 0.001 |
| MUAC1, (804) | 24.3±2.94 | 23.2±2.74 | 0.001 |
| MUAC2, (615) | 24.9±2.96 | 23.8±2.69 | 0.001 |
| MUAC3 (563) | 25.1±2.96 | 23.7±2.49 | 0.001 |

Note. Values are Mean ± SD, Figure in parentheses indicate no. of pregnant women;

Wt – weight; MUAC- Mid Upper Arm Circumference

Wt1 and MUAC1- data collected when mean and SD of gestation was 23.6±6.55 weeks

Wt2 and MUAC2- data collected when mean and SD of gestation was 32.6±3.78 weeks

Wt3 and MUAC3- data collected when mean and SD of gestation was 38.5±2.09weeks

SGA- Small for gestation age

**Table 3** Serial cut- off values and validity indices of maternal Wt, Ht and MUAC as an indicator of LBW

| **Wt** | Sensitivity | Specificity | PPV | NPV | Accuracy | AUC | p.value |
| --- | --- | --- | --- | --- | --- | --- | --- |
| 50 | 21.6 | 91.7 | 36.5 | 84.1 | 79.0 | 0.57 | 0.005 |
| 55 | 62.5 | 59.9 | 25.7 | 87.8 | 60.4 | 0.64 | 0.001 |
| 56 | 67.0 | 56.2 | 25.3 | 88.5 | 58.1 | 0.64 | 0.001 |
| 57 | 71.6 | 51.1 | 24.5 | 89.0 | 54.8 | 0.63 | 0.001 |
| 58 | 71.6 | 47.6 | 23.2 | 88.3 | 52.0 | 0.61 | 0.005 |
| **Ht** |  |  |  |  |  |  |  |
| 148 | 25.0 | 81.8 | 23.4 | 83.0 | 71.4 | 0.53 | 0.320 |
| 150 | 39.8 | 70.1 | 22.9 | 83.9 | 64.6 | 0.55 | 0.146 |
| 152 | 56.8 | 56.2 | 22.4 | 85.4 | 56.3 | 0.57 | 0.056 |
| 154 | 69.3 | 41.0 | 20.7 | 85.7 | 46.2 | 0.55 | 0.129 |
| 156 | 80.7 | 27.6 | 19.9 | 86.5 | 37.3 | 0.54 | 0.224 |
| **MUAC** |  |  |  |  |  |  |  |
| 20 | 12.6 | 96.2 | 42.3 | 83.3 | 81.0 | 0.54 | 0.197 |
| 21 | 24.1 | 88.8 | 32.3 | 84.1 | 77.1 | 0.56 | 0.059 |
| 22 | 36.8 | 74.8 | 24.4 | 84.2 | 67.9 | 0.56 | 0.091 |
| 23 | 54.0 | 59.8 | 22.9 | 85.5 | 58.8 | 0.57 | 0.044 |
| 24 | 71.3 | 42.0 | 21.4 | 86.8 | 47.3 | 0.57 | 0.053 |
| 25 | 81.6 | 31.8 | 20.9 | 88.7 | 40.8 | 0.57 | 0.050 |

Note. Wt - weight; Ht- height, MUAC - Mid Upper Arm Circumference (Wt, Ht and MUAC data of 23.6±6.55 weeks of gestation). PPV- positive predictive value, NPV- Negative predictive value and AUC - Area under the curve; LBW - Low birth weight.

**Table 4** Regression analysis of the LBW and SGA neonates on Maternal MUAC1 with different cut-offs

|  | LBW | | | SGA | | |
| --- | --- | --- | --- | --- | --- | --- |
| Independent variable | OR | P value | 95% CI | OR | P value | 95% CI |
| MUAC <24 | 3.405 | 0.006 | 1.43-8.1 | 2.74 | 0.009 | 1.28-5.84 |
| MUAC <23 | 1.083 | 0.86 | 0.46-2.58 | 0.902 | 0.79 | 0.42-1.93 |
| MUAC <22 | 1.54 | 0.59 | 0.47-3.76 | 0.97 | 0.95 | 0.4-2.34 |

Note. LBW- Low birth weight; MUAC-Mid upper arm circumference; SGA- Small for gestational age; OR- odds ratio. Significance at p.value ≤ 0.05.

**Table 5** Pearson’s Correlation coefficients (and P-values) between anthropometric measurements in 485 pregnant woman and their newborn children

|  | Birth weight (g) | | Length (cm) | | HC (cm) | |
| --- | --- | --- | --- | --- | --- | --- |
|  | r | P | r | p | r | p |
| Age | 0.119** | 0.009 | 0.154** | 0.002 | 0.153** | 0.002 |
| Ht | 0.210** | 0.0011 | 0.205** | 0.0011 | 0.186** | 0.001 |
| Wt1 | 0.251** | 0.001 | 0.220** | 0.0011 | 0.196** | 0.001 |
| Wt2 | 0.288** | 0.0011 | 0.253** | 0.0011 | 0.192** | 0.001 |
| Wt3 | 0.316** | 0.0011 | 0.265** | 0.0011 | 0.192** | 0.001 |
| GWG | 0.202** | 0.0011 | 0.138** | 0.005 | -0.001 | 0.99 |
| MUAC1 | 0.199** | 0.0011 | 0.162* | 0.001 | 0.123* | 0.014 |
| MUAC2 | 0.213** | 0.0011 | 0.194** | 0.0011 | 0.142** | 0.005 |
| MUAC3 | 0.253** | 0.0011 | 0.191** | 0.0011 | 0.142** | 0.007 |
| PWT | 0.288** | 0.0011 | 0.220** | 0.0011 | 0.151** | 0.002 |
| GAD | 0.321** | 0.0011 | 0.154** | 0.002 | 0.146** | 0.003 |

Note. Wt - Weight; Ht - Height; MUAC - Mid Upper Arm Circumference; PWt - Post natal weight of women; GWG - Gestational Weight Gain. Length- Crown Heal length; HC- Head Circumference of the neonates.

**Supplementary Table 1** Maternal body composition at different stages of gestation with birth weight, length and HC

|  | | BFPER1 | FATKG1 | LBM1 | BFPER2 | FATKG2 | LBM2 | BFPER3 | FATKG3 | LBM3 |
| --- | --- | --- | --- | --- | --- | --- | --- | --- | --- | --- |
| Length | Pearson Correlation | .055 | .121^**^ | .202^**^ | .088 | .162^**^ | .251^**^ | .087 | .187^**^ | .290^**^ |
|  | Sig. (2-tailed) | .200 | .005 | .001 | .057 | .001 | .001 | .077 | .001 | .001 |
|  | N | 535 | 535 | 535 | 465 | 465 | 465 | 416 | 415 | 415 |
| HC | Pearson Correlation | .080 | .132^**^ | .184^**^ | .098^*^ | .137^**^ | .175^**^ | .079 | .157^**^ | .232^**^ |
|  | Sig. (2-tailed) | .066 | .002 | .001 | .036 | .003 | .001 | .109 | .001 | .001 |
|  | N | 528 | 528 | 528 | 460 | 460 | 460 | 410 | 409 | 409 |
| Bwt | Pearson Correlation | .111^**^ | .169^**^ | .236^**^ | .146^**^ | .211^**^ | .291^**^ | .158^**^ | .262^**^ | .352^**^ |
|  | Sig. (2-tailed) | .003 | .001 | .001 | .001 | .001 | .001 | .001 | .001 | .001 |
|  | N | 733 | 733 | 733 | 590 | 590 | 590 | 500 | 499 | 499 |

Note. * Correlation is significant at the 0.05 level (2-tailed). ** Correlation is significant at the 0.01 level (2-tailed).


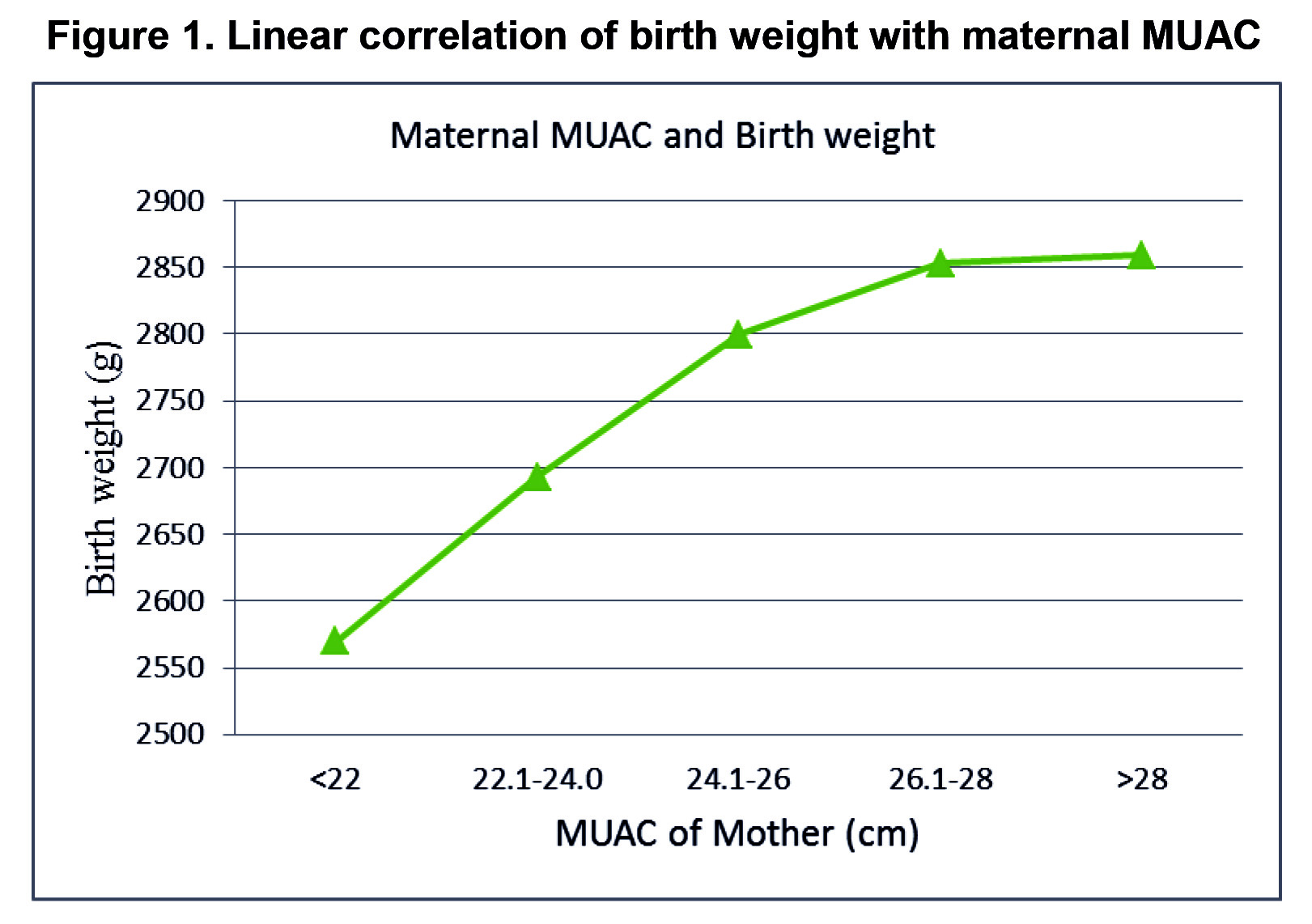

Supplement: Supplementary file 1 — Table S1. Maternal body composition at different stages of gestation with birth weight, length and HC [file MCN-16-e12902-s001.docx]
